# Retraction: Silent Myocardial Infarction: A Case Report

**DOI:** 10.7759/cureus.r165

**Published:** 2025-01-28

**Authors:** Maria V Kolesova, Suzanne Minor

**Affiliations:** 1 Medical School, Florida International University, Herbert Wertheim College of Medicine, Miami, USA; 2 Office of Academic Affairs, Florida International University, Miami, USA

This article has been retracted by the Editors-in-Chief due to the following errors with the ECG (Figure 1). The caption does not make sense for the ECG shown. The arrows do not point to what is stated in the caption. There are no yellow arrows as stated in the caption. The blue arrows do not point to a QRS, and the green arrows do not point to Q waves. The ECG is the focal point of the article, but it does not actually represent any of the characteristics described within. Repeated attempts to contact the authors have been unsuccessful. As a result, the journal is left with no choice but to retract this article.

